# The analysis of MSTMOVCF (Multi-segment thoracolumbar mild osteoporotic fractures surgery or conservative treatment) based on ASTLOF (the assessment system of thoracolumbar osteoporotic fracture)

**DOI:** 10.1038/s41598-018-26562-7

**Published:** 2018-05-29

**Authors:** Jin Peng Du, Yong Fan, Ji Jun Liu, Jia Nan Zhang, Yan Sheng Huang, Jing Zhang, Ding Jun Hao

**Affiliations:** 1 Department of Spine Surgery, Xi’an Jiao Tong University-affiliated Hong Hui Hospital, Youyidong Road, Xi’an City, 710000 Shaanxi Province China; 2 Medical College, Yan’an University, No 38 Guanghua Road, Yan’an City, 716000 Shaanxi Province China

## Abstract

To investigate the issue that conservative or surgical treatment for multi-segmental thoracolumbar mild osteoporotic vertebral compression fracture (MSTMOVCF) by applying the assessment system of thoracolumbar osteoporotic fracture (ASTLOF). A single-center prospective cohort study was designed to enroll elderly patients with MSTMOVCF from June 2013 to June 2016, which were divided into conservative and surgery group. The primary outcomes were Visual Analogue Scale (VAS) score and Oswestry Disability Index (ODI) score, with secondary outcomes including SF-36 and imaging measures such as height of anterior and middle column, Beck value, complications. A total of 470 patients with MSTMOVCF were enrolled. 193 patients underwent surgery of percutaneous vertebroplasty (PVP) or percutaneous kyphoplasty (PKP) and 277 patients underwent conservative treatment. The VAS score of operation group was significantly lower than that of conservative group (P < 0.0001, for all). The ODI score of the operation group was significantly lower than that of conservative group (P < 0.0001, for all). The SF-36 score, height of anterior and middle column, Beck value in the operation group were higher than those in conservative group (P < 0.0001, for all) at 1-year follow-up. MSTMOVCF underwent surgery can achieve great short-term clinical results. The patient with the sum of revised ASTLOF scores of multiple injured vertebrae ≥ 5 was recommended for surgery.

## Introduction

Osteoporotic vertebral compression fractures (OVCF) are more common in the elderly^1^, it often caused by low-energy damage. Improper treatment will not only affect the stability and balance of the spine, severe cases can lead to neurological damage, increase the risk of death^2^. The classification of OVCF includes Chinese Medical Association classification (compression type, burst type), Genant semi-quantitative method and Heini classification^3,4^. These classifications are mainly based on fracture morphology characteristic and (or) stability present on X-ray films, but as a result of evaluation index is single, the severity of osteoporotic fracture can’t be assessed, which will inevitably affect the treatment and prognosis of fractures. Even now there are still many spinal surgeons based on the TLICS score to determine osteoporotic fractures require surgery or not, but which is wrong, because the TLICS score is primarily used for thoracolumbar fractures caused by high energy damage. For these reasons, our team put forward a classification system (The assessment system of thoracolumbar osteoporotic fracture, ASTLOF) that can fully reflect the severity of OVCF based on previous studies and combined its own characteristics (morphological changes, bone mineral density, MRI manifestation and clinical symptoms)^5^. It has been verified that the system has high consistency and repeatability, simple to use, comprehensive and accurate assessment, which can effectively guide the treatment of thoracolumbar OVCF^6^. However, it is difficult to answer the question that conservative or surgical treatment for multi-segmental thoracolumbar mild osteoporosis compression fracture (MSTMOVCF) by applying this system. The purpose of this study is to address this issue and to revise our classification system to better guide clinical practice.

## Methods

**Figure 1:**
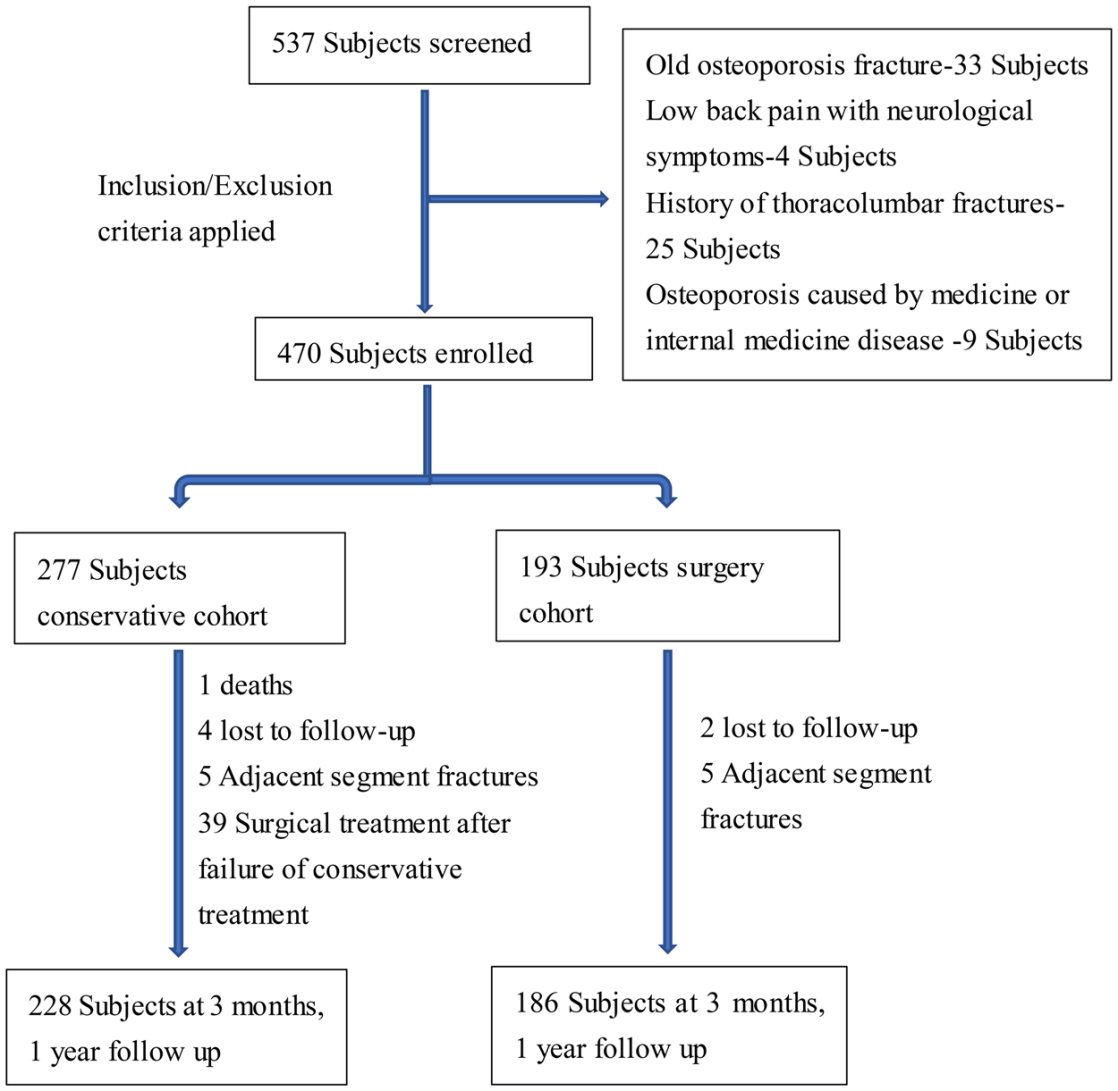
Diagram showing the process of patient selection.

A single-center prospective cohort study was designed to enroll elderly patients with MSTMOVCF from June 2013 to June 2016 after the approval of the Honghui Hospital Ethics Committee, informed consent was obtained from all included subjects. Inclusion criteria: (1) meet the diagnostic criteria of osteoporosis (WHO recommended criteria for diagnosis and treatment of osteoporosis); (2) MRI confirmed the existence of multi-segment fresh thoracolumbar fractures; (3) symptoms of thoracolumbar back pain, no neurological symptoms; (4) according to the classification, MSTMOVCF means mild vertebral collapse and are defined as ASTLOF score for the most severe injured segment ≤4, the sum of multi-segmental ASTLOF score ≥5. Exclusion criteria: (1) history of previous fractures; (2) causes of violence; (3) osteoporosis caused by drugs or internal diseases; (4) pathologic fracture caused by spinal primary tumor or metastatic tumor; (5) infectious diseases. The flow chart of screening is shown in Fig. 1.

Patients eligible for enrollment after screening were non-randomly assigned to either conservative cohort or surgical cohort based on the patient’s vital signs can tolerate surgery or not, patient’s willingness to surgery and the requirement for quality of life. ASTLOF includes four aspects: morphological change of the injured vertebra, MRI manifestations of the injured vertebra, bone mineral density and clinical manifestations (pain/neurological symptoms). According to different situations to give different score (Table 1), and finally add the four parts, the total score can be used as the basis for the choice of treatment. Total score ≤ 3, non-surgical treatment was recommended: bed-rest + anti-osteoporosis drugs + brace + analgesic drugs; Total score = 4, non-surgical treatment or surgical treatment (vertebroplasty or kyphoplasty) should be based on the patient’s vital signs can tolerate surgery or not, patient’s willingness to surgery and the requirement for quality of life; Total score ≥5, require surgical treatment: percutaneous vertebroplasty (PVP), percutaneous kyphoplasty (PKP) or open surgery. The modified ASTLOF was used in this study. The total ASTLOF score was equal to the sum of score of the most serious vertebra and score of morphological changes and MRI manifestations of the other injured vertebra. Total score <5, advise conservative treatment, total score ≥5, advise surgical treatment.

**Table 1:** The assessment system of thoracolumbar osteoporotic fracture (ASTLOF).

Characteristic Value	
Morphology	
Normal	0
Compression fractures	1
Burst fracture	2
MRI	
Normal	0
Long T1 and T2 signal	1
Vacuum or effusion in vertebral body	2
Bone mineral density	
T > −2.5	0
−2.5 > T > −3.5	1
T < −3.5	2
Pain	
No obvious pain	0
Back and lower back pain	1
Sustained pain or spinal cord injury	2
Total score	0–8

ASTLOF was put forward in the present study in order to guide the treatment of thoracolumbar osteoporotic fractures.

Conservative treatment programs are bed rest + brace + anti-osteoporosis (calcium, calcitriol, calcitonin) and analgesic therapy, which are used in strict accordance with the recommendations of the Guidelines to diagnosis and treatment of osteoporotic fracture in China^7^. The surgical treatment was PKP/PVP, the first day after surgery under the brace protection to get up for activities. To avoid leakage of bone cement during surgery, if which is found to cause neurological symptoms, and then emergency treatment for spinal canal decompression. All patients underwent surgery were treated with anti-osteoporosis treatment and short-term brace protection to reduce the incidence of adjacent-segment fracture.

Patients enrolled in the study whose VAS (visual analogue scale) score, ODI (The Oswestry Disability Index) score, SF-36 (The MOS item short from health survey) value include PCS (Physical component summary) and MCS (Mental component summary) within 12 hours after admission were collected, and general characteristics of patients such as gender, age, BMI, duration of symptoms were also recorded. The X-ray, CT, MRI and bone mineral density were examined, the height of anterior and middle column (VAH and VMH) and the Beck value (the ratio of anterior-posterior height of vertebral body) were measured. To determine bone mineral density value, bone mineral density check was performed with dual-energy X-ray absorption method (DXA) according to WHO diagnostic criteria. The assessment of the ASTLOF score was made independently by two senior doctors (Ji Jun Liu and Yong Fan) who are familiar with the system and blind to the group allocation, consensus was achieved after discrepancies were resolved through discussion between them.

The primary outcomes of this study were VAS score and ODI score at post-treatment 2 days, 3 months, 1-year follow-up (Post-2D, Post-3M, Post-1Y). Calculate the improvement rate of ODI [improvement rate (%) = (1 – post-treatment ODI/pretherapeutic ODI) × 100%]. An improvement rate >75% was considered excellent; 50% ~ 75%, was good; 25% ~ 49%, fair; and <25%, poor. Secondary outcomes including health status assessment (SF-36) and imaging measures such as VAH and VM, Beck value, complications. Regular follow-up and data recording by an independent research assistant, relevant data will not be included in the analysis for patients who have not completed the follow-up due to lost follow-up, death, re-fracture, or re-operation.

All methods were performed in accordance with STROBE Statement guidelines.

## Statistical analysis

The Student t-test were used for statistical analysis of continuous variables, if the two groups are not equal in variance, then preferred Stterthwaite. Mean values are presented as the mean ± SD and 95% confidence interval. For quantitative data the chi-square test was used. Significance level was set α  = 0.05. All statistical analyses were performed using Statistical Product and Service Solution Version 18.0 (SPSS, Inc, Chicago, IL, USA).

## Results

From June 2013 to June 2016, the Center treated 537 patients with MSTMOVCF (ASTLOF score for the most severe injured segment ≤ 4, the sum of multi-segmental ASTLOF score ≥ 5). After screening, only 470 patients were eventually enrolled in the study, with an average age of 71.0 ± 10.6 years, of whom 352 were women (74.9%). 277 cases were included in the conservative group, with an average age of 71.5 ± 11.3 years, of whom 205 (74.0%) were women; 193 were included in the operation group, with an average age of 69.7 ± 9.9 years, of whom 147 (76.2%) were women. There were no significant differences between the conservative group and the surgical group in terms of sex, age, BMI, the number of fractured vertebrae, the proportion of noncontignous thoracolumbar fractures. The duration of symptoms in the conservative group was longer than that in the surgical group (P < 0.0001), however, only patients with fresh fractures presented on MRI could be included and such difference did not have a significant effect on the final outcomes. Overall, the baseline data between two groups was basically homogeneous, as shown in Table 2.

**Table 2:** Patients’ demographic data.

Parameter	Conservative	Surgery	P values
Number of patients	277	193	
Age (years)	71.5 ± 11.3	69.7 ± 9.9	0.075
Male (%)	74.0	76.2	0.595
Mean BMI (kg/m2)	25.1 ± 3.6	25.7 ± 3.5	0.073
Symptom duration (days)	18.8 ± 17.4	9.2 ± 8.5	<**0.0001**
Noncontiguous thoracolumbar fracture (%)	46.9	43.5	0.465
Number of injured vertebrae	540	377	
Number of injured vertebrae per case	2.4 ± 0.7	2.3 ± 0.6	0.098
Morphology			0.681
Number of compression fractures (%)	330 (61.1)	222 (58.9)	
Number of burst fracture	74	59	
Bone mineral density (T value)	−(2.8 ± 0.6)	−(3.0 ± 0.6)	0.060

All patients were followed up at the third month and one year after treatment. In the conservative group, 1 patient died and 4 patients lost to follow-up, 5 patients had adjacent-segment fracture and 39 cases of reoperation for conservative treatment failure, in which 13 cases underwent surgery because of bed-rest relevant complications that patient can’t continue to lie in bed or symptoms after conservative treatment failed to relieve obviously. 26 cases of injured vertebral height lost again with thoracolumbar kyphosis, lumbar back stubborn pain, seriously affecting the quality of life, the MRI indicated that the fracture was not healed and the operation had to be performed, the rest of the patients by conservative treatment to achieve bone healing. Finally, only 228 patients in the conservative group completed follow-up and collected all data. In the operation group, 2 patients were lost to follow-up, 5 patients had adjacent-segment fracture and treated with secondary surgery, only 186 patients completed the entire research.

The injured vertebral segment was 917 from T8- L5, with a total of 308 cases of 2 injured segment and 162 cases of 3 injured segment. In the conservative group, 540 injured vertebrae were found, with an average of 2.4 ± 0.7 per case, 330 (61.1%) vertebral bodies were compressed fracture and the mean bone mineral density was - (2.8 ± 0.6). There were 377 injured vertebrae in the operation group, with an average of 2.3 ± 0.6 per case. 222 (58.9%) segments were compressed fracture and the mean bone mineral density was -(3.0 ± 0.7). The above data between the two groups had no significant difference (P > 0.05).

### VAS score and ODI score at follow-up

VAS score and ODI score were evaluated in both groups at 3 months and 1 year after treatment, which were assessed immediately in the operation group 2 days after surgery. The detailed data are shown in Tables 3 and 4. The VAS score for surgery group (Post-2D: 3.4 ± 0.9; Post-3M: 2.0 ± 0.6; Post-1Y: 1.8 ± 0.5), for conservative group (Post-3M: 6.4 ± 1.8; Post-1Y: 4.6 ± 1.3). The ODI score for surgery group (Post-2D: 44.8 ± 5.1; Post-3M: 36.5 ± 4.7; Post-1Y: 30.2 ± 3.4), for conservative group (Post-3M: 60.1 ± 6.8: Post-1Y: 43.6 ± 4.4). There was no significant difference in VAS and ODI score between two groups before treatment (P > 0.1), but a significant difference after treatment (P < 0.05), meanwhile, at the last follow-up, an improvement rate of ODI in the surgery group was 43.6~ 69.5%, mean (60.8 ± 5.7) %, was considered good; in the conservative group was 23.8% ~ 51.6%, mean (41.4 ± 5.3) %, was considered fair. Which means that two methods for thoracolumbar OVCF are all effective. However, after treatment, the score of VAS and ODI in the operation group were significantly lower than those in the conservative treatment group (P < 0.0001), in particular two days after surgery, which were significantly lower than pre-operation, indicating that the PKP/PVP have the advantages of significantly relieving pain and improving dysfunction in a short period of time. We also find the VAS score for surgery group decreased by ~0.2 during period of Post-3M to Post-1Y, this may be due to the fact that most of the patients in the surgical group still have non-specific low back pain caused by osteoporosis after surgery, and anti-osteoporosis treatment is a long-term and slow process. However, Patients in the conservative group were treated for conservative treatment for three months with fresh fractures became old fractures and began walking exercises and functional exercises of the back muscles. At the same time, the patients continued to underwent anti-osteoporosis treatment. During the period from 3 months to 1 year after surgery, the back pain was significantly relieved (VAS score decreased by 1.8), but due to the further collapse of multiple fractured vertebral bodies, the sagittal alignment of spine was changed, such as kyphosis, which caused chronic low back pain. Therefore, patients in the conservative group had more VAS score of low back pain at 1-year follow-up than the surgery group, and the long-term efficacy was worse than that of the surgery group.

**Table 3:** Comparison of VAS score between surgery group and conservative group.

Groups	N	Pre-treatment	Post-2D	Post-3M	Post-1Y
Conservative	228	7.1 ± 1.8 (3.57, 10.63)*	—	6.4 ± 1.8^Δ^ (2.87, 9.93)*	4.6 ± 1.3^Δ^ (2.05, 7.15)*
Surgery	186	7.0 ± 2.0 (3.08, 10.92)*	3.4 ± 0.9^Δ^ (1.64, 5.16)*	2.0 ± 0.6^Δ^ (0.82, 3.18)*	1.8 ± 0.5^Δ^ (0.82, 2.78)*
t value	—	0.535	—	34.627	29.922
P value	—	0.593	—	<**0.0001**	<**0.0001**

*95% Confidence interval (CI). ^Δ^There is a significant difference compared with the pre-treatment, P < 0.05. VAS: Visual analogue scale.

**Table 4:** Comparison of ODI score between surgery group and conservative group.

Groups	N	Pre-treatment	Post-2D	Post-3M	Post-1Y
Conservative	228	69.6 ± 7.5 (54.90, 84.30)*	—	60.1 ± 6.8^Δ^ (46.77, 73.43)*	43.6 ± 4.4^Δ^ (34.98, 52.22)*
Surgery	186	70.1 ± 6.9 (56.58, 83.62)*	44.8 ± 5.1^Δ^ (34.80, 54.80)*	36.5 ± 4.7^Δ^ (27.29, 45.71)*	30.2 ± 3.4^Δ^ (23.54, 36.86)*
t value	—	0.699	—	41.617	34.942
P value	—	0.485	—	<0.0001	<0.0001

*95% Confidence interval (CI). ^Δ^There is a significant difference compared with the pre-treatment, P < 0.05. ODI: Oswestry disability index.

### The outcomes of the secondary endpoints

The patients underwent SF-36 evaluation and review of the X-ray film during the one-year follow-up. VAH, VMH and the Beck value were measured. The detailed data are shown in Tables 5 and 6. SF-36 score for surgery group (Pre: PCS 26.5 ± 7.0; MCS 28.8 ± 10.4; Post-1Y: PCS 48.6 ± 10.4; MCS 44.9 ± 12.9), conservative group (Pre: 27.4 ± 7.2; MCS 26.7 ± 9.6; Post- 1Y: PCS 37.6 ± 8.8; MCS 39.0 ± 12.1). There was no significant difference in secondary outcomes between the two groups before treatment, except for the MCS in SF-36. We found that the MCS was significantly lower in the conservative group than that of in the surgical group before treatment (P = 0.034), possibly due to patients with longer duration of symptoms in the conservative group, most underwent conservative treatment by themselves at home but symptoms still cannot be significantly alleviated, caused a mental depression and anxiety. SF-36 score in both groups after treatment were higher than that before treatment (P < 0.05), and operation group was significantly higher than conservative group (P < 0.0001). The height of anterior and middle column, Beck value for conservative group (VAH: 18.0 ± 3.6; VMH: 16.7 ± 3.5; Beck: 0.71 ± 0.18) and for surgery group (VAH: 21.4 ± 4.4; VMH: 19.8 ± 4.1; Beck: 0.83 ± 0.21) at 1year follow-up. After treatment, VAH and VMH, Beck values in conservative group were lower than pre-treatment (P < 0.05), suggesting that generally vertebral collapse and vertebral Cobb angle increased. After PKP/PVP treatment of fractured vertebrae, VAH, VMH and Beck value were significantly increased than pre-operation (P < 0.05). VAH increased by an average of 2.5 mm and VMH increased by an average of 2.0 mm. 130 patients (67.4%) were treated with PKP in the operation group, suggesting that a good reduction effect may be related to the treatment of more patients with PKP. Obviously, VAH, VMH and Beck value in the injured vertebrae of the operation group were significantly higher than those of the conservative group at follow-up one year after treatment (P < 0.0001).

**Table 5:** Comparison of SF-36 score between surgery group and conservative group.

Groups	N	Pre-treatment		Post-1Y	
		PCS	MCS	PCS	MCS
Conservative	228	27.4 ± 7.2 (13.29, 41.51)*	26.7 ± 9.6 (7.88, 45.52)*	37.6 ± 8.8^Δ^ (20.35, 54.85)*	39.0 ± 12.1^Δ^ (15.28, 62.72)*
Surgery	186	26.5 ± 7.0 (12.78, 40.22)*	28.8 ± 10.4 (8.42, 49.18)*	48.6 ± 10.4^Δ^ (28.22, 68.98)*	44.9 ± 12.9^Δ^ (19.62, 70.18)*
t value	—	1.281	2.132	11.461	4.790
P value	—	0.201	**0.034**	<**0.0001**	<**0.0001**

^*^95% Confidence interval (CI). ^Δ^There is a significant difference compared with the pre-treatment, P < 0.05. PCS: Physical component summary; MCS: Mental component summary.

**Table 6:** Comparison of VAH, VMH and Beck value between surgery group and conservative group.

Groups	N	Pre-treatment			Post-1Y		
		VAH	VMH	Beck	VAH	VMH	Beck
Conservative	228	19.1 ± 4.1 (11.06, 27.14)*	18.3 ± 3.9 (10.66, 25.94)*	0.77 ± 0.20 (0.38, 1.16)*	18.0 ± 3.6^Δ^ (10.94, 25.06)*	16.7 ± 3.5^Δ^ (9.84, 23.56)*	0.71 ± 0.18^Δ^ (0.36, 1.06)*
Surgery	186	18.9 ± 4.0 (11.06, 26.74)*	17.8 ± 3.8 (10.35, 25.25)*	0.76 ± 0.18 (0.41, 1.11)*	21.4 ± 4.4^Δ^ (12.78, 30.02)*	19.8 ± 4.1^Δ^ (11.76, 27.84)*	0.83 ± 0.21^Δ^ (0.42, 1.24)*
t value	—	0.499	1.313	0.529	8.475	8.166	6.162
P value	—	0.618	0.190	0.597	<**0.0001**	<**0.0001**	<**0.0001**

*95% Confidence interval (CI). ^Δ^There is a significant difference compared with the pre-treatment, P < 0.05. VAH: vertebral anterior height; VMH: vertebral middle height.

### Complications

**Figure 2:**
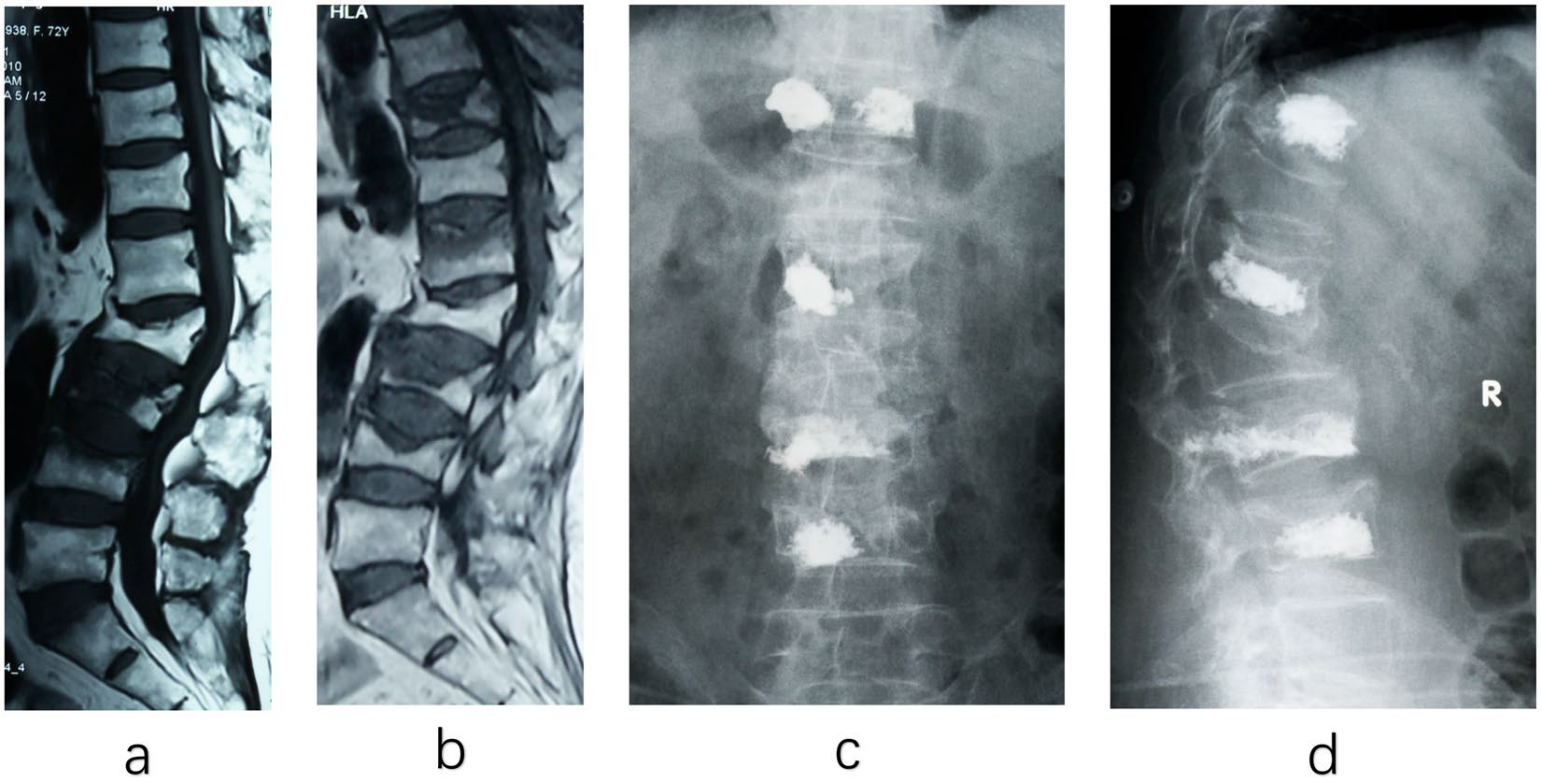
A 70-year-old female patient, thoracolumbar multi-segment osteoporotic fracture, conservative treatment failed with adjacent-segment fractures and then be treated surgically. (**a**) First admission (2014–06–17): MRI showed lumbar compression fracture in L3, 4 and old lumbar compression fracture in L2. L3 ASTLOF score of 4 points, the total ASTLOF score of 6 points, underwent conservative treatment. (**b**) The second admission (2014–10–14): MRI showed vertebral collapse in L3 and L4 compared with previous MRI, new thoracolumbar compression fracture in T11 and L1. T11 ASTLOF score of 4 points, the total ASTLOF score of 10 points, underwent PKP. (**c,d**) Postoperative review of the X-ray showed: bone cement leakage in T11 and L3, no complications were found, and back pain were relieved.

In the conservative group, bed-related complications occurred in 21 (9.2%) patients, including 5 cases of hypostatic pneumonia, 2 cases of urinary tract infection, 13 cases of bedsore and 1 case of pulmonary embolism. 5 (2.2%) patients had adjacent-segment fracture. 26 (11.4%) cases of injured vertebral height again lost with thoracolumbar kyphosis, lumbar back stubborn pain. See Fig. 2 for typical case. There were 39 (20.9%) cases of cement leakage in the operation group, but none of them showed neurological symptoms. adjacent-segment fractures occurred in 5 (2.6%) cases, of which 1 had neurological symptoms and had to underwent surgical decompression. There was no significant difference in the incidence of adjacent-segment re-fracture between two groups (P > 0.1).

## Discussion

For patients with osteoporosis, minor trauma can often lead to compression fractures of one or more vertebral bodies, partial patients may not have a clear history of trauma, the main clinical manifestation is back/low back pain (height reduction and kyphosis deformity, and the degree of pain individuals big difference). At present, the classification of thoracolumbar fractures is mainly classified according to spinal morphological changes and stability, such as Denis classification, AO classification. Vaccaro *et al*. put forward the Thoracolumbar Injury Classification and Severity Score (TLICS)^8^, which has the greatest advantage of incorporating neurologic status and the integrity of the posterior ligamentous complex (PLC) into the classification, which can be more fully evaluate the severity of fractures. These systems are mainly focused on high-energy violence injury, while the thoracolumbar OVCF is mainly characterized by low-energy damage and osteoporosis. The previous mentioned classifications did not take into account the particularity of OVCF and it was obviously not suitable for applying to the classification of OVCF. Currently it still lacks a comprehensive and systematic assessment of the severity of bone fracture which could provide clinical guidance for treating thoracolumbar OVCF. Recently, our team put forward a classification system (ASTLOF) for OVCF based on previous studies and combined its own characteristics (morphological changes, bone mineral density, MRI manifestation and clinical symptoms)^5^, and recommended the corresponding treatment plan according to different scores. The system can predict the severity of fractures while diagnosing osteoporotic fractures. Its reliability, including consistency and repeatability, has been proved, and the ASTLOF method appeared to be helpful in guiding clinical practice.

The controversy over the use of PVP/PKP in the treatment of OVCF has subsided in recent years as a large number of high-quality studies, including RCT and meta-analysis, have shown that PVP/PKP is superior to conservative treatment both in the short and long term^2,9^. Even many studies supporting multi-segment OVCF treated with PKP/PVP have not only proven great clinical outcomes, but also have a lower risk of neurological symptoms caused by cement leakage and a lower risk of re-fractures of adjacent segments^10,11^. Therefore, MSMTOVCF can also be radically treated with surgery, or still only need conservative treatment, this issue has not been studied.

The current research answered this question through an improved ASTLOF. After research we found that primary and secondary results all support surgery was significantly better than conservative treatment. In particular, short-term VAS score and ODI score decreased obviously after surgery, long-term follow-up showed the effect is stable. It found that patients with generally vertebral collapse and vertebral Cobb angle increased at 1-year follow-up after conservative treatment, meanwhile, the operation therapy indicating great reduction effect of injured vertebra, no case of collapse of the vertebra with bone cement. In terms of complications, there was no significant difference in the incidence of adjacent-segment re-fractures between two groups, while in the conservative group, there was a markedly higher bed-related complication, 11.4% cases of injured vertebral height lost again with thoracolumbar kyphosis, lumbar back stubborn pain. Although better short-term clinical results in the surgery group, there was a higher rate of cement leakage and the risk of neurological symptoms caused by cement leakage. In summary, we recommend to improve the ASTLOF into two parts, the first part of the score equal to the sum of score for multiple injured vertebral morphology changes and MRI manifestation, the second part is the sum of score of pain and bone mineral density examination. The sum of score of two parts ≥5, it is recommended that surgical treatment (vertebroplasty, kyphoplasty or open surgery) and the total score of two parts <5, which is recommended for conservative treatment. See Table 7 for details.

**Table 7:** The improved version of assessment system of thoracolumbar osteoporotic fracture (ASTLOF).

Characteristic	Value
**Part 1**	
Morphology (N = n_1_ + n_2_ + n_3_)	
Normal (n_1_)	0xn_1_
Compression fractures (n_2_)	1xn_2_
Burst fracture (n_3_)	2xn_3_
MRI (N = n_a_ + n_b_ + n_c_)	
Normal (n_a_)	0xn_a_
Long T1 and T2 signal (n_b_)	1xn_b_
Vacuum or effusion in vertebral body (n_c_)	2xn_c_
**Part 2**	
Bone mineral density	
T > −2.5	0
−2.5 > T > −3.5	1
T < −3.5	2
Pain	
No obvious pain	0
Back and lower back pain	1
Sustained pain or spinal cord injury	2
Total score	Part 1 + Part 2

N = n_1_ + n_2_ + n_3_ means that the sum of number of injured vertebra present in normal, compression fractures and burst fracture is equal to the total number of injured vertebra.

N = n_a_ + n_b_ + n_c_ means that the sum of number of injured vertebra present in normal, long T1 and T2 signal and vacuum or effusion in vertebral body is equal to the total number of injured vertebra.

ASTLOF was put forward in the present study in order to guide the treatment of thoracolumbar osteoporotic fractures.

Surgeons should also consider the patient’s adherence to the treatment program when choosing a specific therapy. In our experience, elderly patients are often not able to strictly perform medical advice during their conservative treatment. They usually start prematurely activities under the condition of partial relief of pain after the acute stage of fracture but not yet enough to 3 months for bed-rest, or even reject to wear the brace because of uncomfortable feeling, which may be reasons why the vertebral height highly prone to collapse, thoracolumbar kyphosis deformity, chronic low back pain, then leading to failure of conservative treatment. In addition, conservative treatment for bedridden patients requires special support, patients’ children are busy with work, a lot of time costs and better medical insurance policies in China make them prefer to choose surgical treatment in order to obtain rapid surgical effects and avoid the burden of life caused by conservative treatment.

At the time of surgery, the strategy is to treat all the injured vertebrae, or only to deal with the injured vertebra that is mainly responsible for the pain, there are still controversial^12,13^. According to our experience, it is difficult to identify the source of pain when there are multiple injured vertebrae, surgical treatment only for the most severe segment but with a risk of postoperative insignificant pain relief, we are used to apply a large number of bone cement injection to serious injured segment to relieve the pain and strengthen fixation, and for the vertebral body of mild fractures with a small amount of cement injection to relieve the pain. Therefore, the surgery group all used this method in current study. Some studies have shown that unilateral bone cement injection and bilateral cement injection in the clinical outcomes of no significant difference^14–16^, so we most used a unilateral injection method. In addition, the incidence of neurological symptoms caused by cement leakage in this study is 0% compared with the previous literature and may be related to the following surgical techniques^17^: 1. Perform the operation under high-definition fluoroscopy in the DSA room; 2. To avoid for pursuing great dispersion effects, injected under the condition of soft cement; 3.Using minor skills such as multiple times injection, each time a small amount of injection and timely adjustment approach to avoid a lot of bone cement leakage.

Studies have shown that alendronate can significantly inhibit bone resorption, increase bone mineral density and reduce the risk of fracture, in particular, has a clear effect to reduce the incidence of vertebral fractures^18^. The bone cement can provide immediate postoperative stability after Vertebroplasty, and bone cement is not absorbable nor has boned-induced effect, without waiting for bone healing, so we recommend that early take bisphosphonates of anti-osteoporosis drugs to reduce the incidence of further fractures.

There are still some limitations in this study, which may have a slight impact on the conclusion of the study because there are several cases in the conservative group who have failed to collect the complete data due to the reoperation for conservative failure. In addition, most of the patients who underwent surgical treatment were treated with PKP, the clinical effects of PKP and PVP may be different, the current study also failed to address the impact of this confounding factor. Improved ASTLOF its consistency and repeatability need further study in order to better guide clinical practice.

## Conclusions

Multi-segment thoracolumbar mild osteoporotic fracture underwent surgery can achieve great short-term clinical results. The patients with the sum of revised ASTLOF scores of multiple injured vertebrae ≥5 was recommended for surgery. The consistency and repeatability of the revised ASTLOF need further study.
